# Prevalence and correlates of men’s and women’s alcohol use in agrarian, trading and fishing communities in Rakai, Uganda

**DOI:** 10.1371/journal.pone.0240796

**Published:** 2020-10-30

**Authors:** Jennifer A. Wagman, Dorean Nabukalu, Amanda P. Miller, Maria J. Wawer, Robert Ssekubugu, Hadijja Nakowooya, Betty Nantume, Eunhee Park, Judith A. Hahn, David M. Serwadda, Nelson K. Sewankambo, Fred Nalugoda, Godfrey Kigozi

**Affiliations:** 1 Department of Community Health Sciences, Jonathan and Karin Fielding School of Public Health, University of California Los Angeles, Los Angeles, California, United States of America; 2 Rakai Health Sciences Program / Uganda Virus Research Institute, Kalisizo, Uganda; 3 Division of Infectious Diseases and Global Public Health, Department of Medicine, University of California San Diego, School of Medicine, La Jolla, California, United States of America; 4 Department of Epidemiology, Johns Hopkins Bloomberg School of Public Health, Baltimore, Maryland, United States of America; 5 Division of HIV, Infectious Diseases, and Global Medicine, Department of Medicine, University of California San Francisco School of Medicine, San Francisco, California, United States of America; 6 Makerere University School of Public Health, Kampala, Uganda; 7 Makerere University School of Medicine, Kampala, Uganda; University of the Witwatersrand, SOUTH AFRICA

## Abstract

**Introduction:**

Uganda has one of the highest rates of alcohol use in sub-Saharan Africa but prevalence and correlates of drinking are undocumented in the Rakai region, one of the earliest epicenters of the HIV/AIDS epidemic in East Africa.

**Methods:**

We analyzed cross-sectional data from 18,700 persons (8,690 men, 10,010 women) aged 15–49 years, living in agrarian, trading and fishing communities and participating in the Rakai Community Cohort Study (RCCS) between March 2015 and September 2016. Logistic regression models assessed associations between past year alcohol use and sociodemographic characteristics, other drug use and HIV status, controlling for age, religion, education, occupation, marital status, and household socioeconomic status.

**Results:**

Past year alcohol prevalence was 45%. Odds of drinking were significantly higher in men (versus women) and fishing communities (versus agrarian areas). Odds of drinking increased with age, previous (versus current) marriage and past year drug use. By occupation, highest odds of drinking were among fishermen and (in women) bar/restaurant workers. Alcohol-related consequences were more commonly reported by male (vs. females) drinkers, among whom up to 35% reported alcohol dependence symptoms (e.g., unsteady gait). HIV status was strongly associated with alcohol use in unadjusted but not adjusted models.

**Conclusions:**

Alcohol use differed by gender, community type and occupation. Being male, living in a fishing community and working as a fisherman or restaurant/bar worker (among women) were associated with higher odds of drinking. Alcohol reduction programs should be implemented in Uganda’s fishing communities and among people working in high risk occupations (e.g., fishermen and restaurant/bar workers).

## Introduction

Alcohol consumption was the leading global risk factor for death among people 15–49 years in 2016, with 3.8% of female and 12.2% of male deaths attributable to its use [1]. It has been found, worldwide, that men consume more alcohol than women, but women who drink are at greater risk for a range of adverse health outcomes [1, 2]. These findings highlight the importance of understanding gender differences in patterns of and risk factors for alcohol use and developing gender-specific prevention and treatment approaches.

Prevalence of alcohol use in all people is greatest in high Socio-Demographic Index locations (e.g., Canada, the United States), but low Socio-Demographic Index locations (e.g., Haiti, Papua New Guinea, Uganda) are more negatively impacted by alcohol’s adverse effects [1]. According to the World Health Organization’s (WHO) 2018 Global Status Report on Alcohol and Health, Africa had the highest age-standardized alcohol-attributable burden of disease and injury in the world [3].

While the majority of Africans (67.8%) abstain from drinking alcohol, many who *do* drink, consume levels and/or have patterns of use that are hazardous or harmful [3]. Hazardous levels or patterns of drinking refer to use that increases the risk of harmful (physical, mental or social) consequences for the drinker or other people [4, 5]. Hazardous levels can be measured in two ways—via total alcohol per capita consumption (APC) and total APC among drinkers. Both measures are assessed in terms of liters of pure alcohol per calendar year. In practice, hazardous drinking patterns are measured by assessing what is referred to as ‘heavy episodic drinking’ (also called ‘binge drinking’), which is defined as the consumption of 60 or more grams of pure alcohol on at least one single occasion in the past 30 days. Hazardous alcohol use is considered ‘risky’ drinking because people with this pattern of consumption are at heightened likelihood for advancing to the category of harmful drinking patterns, if alcohol screening and intervention are not introduced [4, 5]. Harmful drinking refers to use that results in clear evidence of negative consequences that are social (e.g., loss of employment) and/or physical (e.g., hypertensive heart disease) and/or mental health (e.g., depression and anxiety) in nature [4, 5]. Harmful drinking can be measured by assessing alcohol–attributable fractions for deaths from all causes or alcohol use disorders [3–5]. Both hazardous and harmful drinking can lead to alcohol dependence, which refers to a grouping of behavioral, cognitive, and physiological consequences that may develop after repeated alcohol use. While most hazardous and harmful drinkers do not reach a state of dependence, all dependent drinkers engage in hazardous alcohol use for at least some time before reaching the state of dependency [4, 5]. Alcohol use disorders (AUD) include hazardous, harmful and dependent drinking and this grouping of disorders impairs a person’s ability to stop or control their use of alcohol, even in the face of adverse social, occupational, or health consequences [6]. While globally recognized as a cause of serious preventable morbidity and mortality, AUDs have received limited attention in sub-Saharan Africa. An increased understanding of hazardous and harmful alcohol use behaviors, in particular, is warranted since extensive research indicates these less severe AUDs are both more common (relative to alcohol dependency) and more responsive to treatment [4, 5].

Uganda has one of the highest rates of alcohol use in sub-Saharan Africa, with an estimated 2016 total APC of 9.5 liters of pure alcohol among people 15 years and older [3]. This is relative to a total APC of 6.3 liters in the entire WHO African Region. In 2016, most (64%) Ugandans reported “abstaining” from drinking in the past 12 months. The 36% who reported drinking alcohol in the past year indicated an average annual consumption of 26 liters of pure alcohol. This equates to an average daily intake of 56.3 grams of pure alcohol or four daily “standard” drinks (containing ~14 grams of pure alcohol). Available findings suggest Ugandan men have higher average past year alcohol consumption (32.7 liters) than women (12.5 liters) [3, 7]. The prevalence of hazardous or heavy episodic drinking among Ugandan drinkers has been reported as 56.9% (68.8% in men and 32.6% in women) and the prevalence of AUDs as 7.1% (12.4% in men and 1.9% in women) [3].

Much of the literature on alcohol use in sub-Saharan Africa has focused on its relationship with HIV infection, an association documented in multiple studies across the continent, including in Uganda [8]. Alcohol use and HIV infection have been closely examined in fishing communities along Lake Victoria, where HIV prevalence is high and two-thirds of new infections have been attributed to drinking alcohol [9]. In Rakai–the Southwestern Ugandan setting of the current study–we previously found alcohol use before sex to be associated with increased risk for HIV incidence in both men and women [10, 11]. Further, among young women (15–24 years) in Rakai, alcohol use before sex was associated with physical intimate partner violence and sexual coercion, both of which heighten the likelihood of engaging in risky sexual behaviors (e.g., multiple sex partners) which subsequently increases vulnerability for HIV infection [10, 11]. Although HIV prevalence in Rakai has fallen over the past decades to 7.9%, it exceeds the national average of 6.2% [12]. Alcohol-reduction interventions are currently unavailable but have been specifically called for in populations with high HIV and alcohol use prevalence, such as key areas of Rakai [8, 9]. We know there is substantial heterogeneity in HIV prevalence throughout the Rakai region; among the 40 communities participating in the Rakai Community Cohort Study (RCCS), a large longitudinal HIV surveillance study focused on communities in Rakai that experience the highest burden of HIV, HIV prevalence varies from 13% in agrarian and trade communities to 41% in fishing communities [13]. To date, however, the community-level prevalence of and factors associated with alcohol use in Rakai are undocumented.

The objectives of this study were to assess the prevalence and correlates of alcohol use among RCCS participants residing in agrarian, trading, and fishing communities in and around the Rakai region in south-central Uganda in order to inform (1) research priorities, (2) the planning of health prevention and education activities most needed in the different types of communities and (3) targeted service delivery. Given that unique differences have been found in male and female patterns of alcohol use in other global settings, we assessed prevalence and correlates of drinking by gender. It is important to note that we focused on understanding patterns of hazardous and harmful alcohol use, versus alcohol dependency, because these are the most common forms of consumption. Further, research indicates hazardous and harmful alcohol use are more effectively intervened upon (relative to dependent drinking) and a goal of our research is to inform evidence-based alcohol reduction programming in the region.

## Materials and methods

### Research setting and study design

The Rakai region is a mostly rural area in southwestern Uganda that borders the United Republic of Tanzania and Lake Victoria. Qualitative research from Rakai suggests commonly consumed types of alcohol include home-made alcohol (also referred to as “local brew and gin,” or *mukomboti*, *mwenge muganda*), commercially brewed beer, and hard liquor, which is often referred to as *waragi enguli*, *or emandule*. The term "waragi" is widely used to refer to locally distilled gin but Uganda Waragi is produced and marketed industrially. Waragi and other hard liquors are commonly sold in small plastic sachets (typically 100ml) called “buveera” or “tot packs” that are manufactured and produced by large-scale distilleries [14].

We conducted a secondary analysis of cross-sectional data collected between March 2015 and September 2016 from the RCCS, an ongoing HIV surveillance cohort initiated in 1994. RCCS surveys are conducted at approximately 18-month intervals among all consenting adults aged 15–49 years. RCCS methods have been described in detail elsewhere [15]. In brief, the RCCS survey includes individuals in 40 communities in and near Rakai District. RCCS communities are categorized as agrarian (n = 27), trading (n = 9) or fishing (n = 4). Agrarian communities are typically far off from the main roads and can be either big or small towns where the most commonly reported primary occupation in the population of RCCS participants is agriculture and/or production and maintenance of crops and farmland. Trading communities are made up of households located in towns with easy access to major roads, where the most commonly reported primary occupation among all RCCS participants is the buying and selling of goods and/or services (e.g., shopkeeper). Trade communities are also characterized by populations with high mobility. Fishing communities refer to residential areas and landing sites on Lake Victoria where the primary occupation of its residents and workers relates to the harvest or processing of fishery resources (e.g., fishing vessel owners, operators, crew; fish processing, fishing) [16].

Prior to each RCCS surveillance survey (including that from which data derive for the current analysis), a census is conducted to identify community members eligible for enrollment. Census procedures include systematically approaching all households within the RCCS communities; recording household GPS coordinates; and enumerating all resident household members by gender, age, relationship to the head of the household, and duration of residence, regardless of whether they are present or absent at the time of the census. Participants who provide written informed consent are interviewed in central community locations (referred to as “hubs”). Eligible individuals not captured at the hubs are approached at their household or place of work to request their participation. Up to two return visits are made in an attempt to enroll eligible participants. The survey collects detailed sociodemographic and behavioral information with questions on sexual behaviors, sexual partners, health status and service utilization, and reproductive health. In the process, free HIV testing services are offered, including provision of HIV results to all consenting participants and couples. Surveys are administered by researchers who are comprehensively trained and provided approximately 6 months of supervised oversight on the administration of the study instrument.

All study instruments and protocols were reviewed and approved by the Western Institutional Review Board (IRB) (which consists of a committee of experts who reviewed and approved the study), the Uganda Virus Research Institute’s Research and Ethics Committee (UVRI-REC) and the Uganda National Council of Science and Technology (UNCST). The plan for analysis and publication of these data was reviewed and approved by the University of California (UC) San Diego Human Research Protections Program (HRPP), the UC Los Angeles HRPP, UVRI-REC and UNCST. All RCCS participants were compensated 10,000 Ugandan shillings (approximately $3 USD at the time of data collection) for their time and transportation refund. To protect participant privacy and anonymity, all data were de-identified prior to analysis.

### Measures

We measured tobacco smoking, defined as “Do you smoke cigarettes, tobacco or a pipe?” (yes/no) and past year drug use, defined as “use of any of the following drugs in the past 12 months: marijuana, amphetamines, aero fuels ("glue"), *mayirungi*, heroin, or something else.” *Mayirungi* is the Luganda word for khat, a plant whose leaves are chewed as a stimulant. We modeled other drug use in the past year two ways—categorically and dichotomously (any drug use/no drug use).

Socio-demographic information was gathered for all participants, including information about age, religion, education level, occupation, marital status and resident community type (agrarian, trading or fishing, as previously described above). Age was a continuous variable that we collapsed into four categories: 15–19 years, 20–24 years, 25–34 years and 35+ years. Marital status was defined using two survey questions “Have you ever been married?” (yes/no) and “Are you currently married” (yes/no). We used responses from these two questions to create a three-level categorical variable: previously married [i.e., being separated, divorced or widowed], never married and currently married. In the survey, religion was defined as “What is your religion?”, with the following response options: none, Catholic, Protestant, Saved/Pentecostal, Muslim and other (specify)”. All Christian denominations were collapsed into a single category and none and other were collapsed due to small cell sizes leaving us with three categories: Muslim, Christian and none/other. Education was defined as “Have you ever gone to school?” (yes/no) and, if yes, “To what level?” which had the following categories: Primary 1–4, Primary 5–7, Secondary 1–4, Secondary 5–6, technical/university, primary professional, O’level professional. We collapsed categories to create a new variable with the two levels: none/primary, and secondary/higher education. Occupation was defined in the survey as “What kind of work do you do, or what kind of activities keep you busy during an average day, whether you get money for them or not?” Participants were free to provide any response and responses were then coded into the appropriate existing category on the survey (e.g. agriculture for home use/barter) or entered into the “other”, specify category if no existing category was a good fit. We collapsed similar response categories and created a new categorical variable with seven categories: agriculture/housework, bar/restaurant, trucking, fishing, student, trade/shopkeeper and other/no occupation. Household socioeconomic status (SES) was determined using information on structure of respondents’ dwelling, based on methods established for use in RCCS analyses and written up previously [17]. HIV infection was diagnosed using a validated three rapid test algorithm, starting with two parallel rapid tests (STAT Pak HIV 1/2, Chembio Diagnostic Systems Inc. and Determine HIV 1/2, Abbott Laboratories, Wiesbaden-Delkeheim, Germany). Discordant results were then reconciled with a third rapid test (Unigold HIV 1/2, Trinity Biotech Plc, Bray, Co. Wicklow, Ireland). Positive samples were confirmed by two serial enzyme immunoassays (Bioelisa HIV-1+2 Ag/A, Biokit S.A., Spain; followed by Murex HIV Ag/Ab, Diasporin S.p. A., Italy) with Western blot confirmation of discordant enzyme immunoassays and for all HIV seroconverters (HIV-1 WB, BioMerieux-Vitek).

The main outcome variable was past year alcohol use, defined as “having drunk any alcohol in the past 12 months, for instance, beer, wine, *waragi* or other spirits, or home-made beer.” To understand consequences as a result of alcohol use among RCCS participants, men and women who reported drinking in the past year were asked the following additional questions:

- To assess alcohol-related aggression and violence: “In the past year, when you drank alcohol, did you get angry? Get violent or into a fight?” (yes/no for each item)
- To assess loss of control related to drinking: “How often during the last year have you felt you should cut down on your drinking or stop altogether?” (Never, occasionally, sometimes, often)
- To assess alcohol’s impact on daily responsibilities and activities: “In the past year, have you ever taken alcohol while you were at work?” (yes/no)
- To assess harmful alcohol use, three measures similar to those included on the World Health Organization’s Alcohol Use Disorders Identification Test (AUDIT) [4, 5] were used to assess:
  ○ **Guilt after drinking:** “In the past year, when you drank alcohol, have you ever have felt ashamed of something that you did while drinking?” (yes/no)
  ○ **Blackouts:** “In the past year, when you drank alcohol, did you ever forget some of the things you did or that happened while you were drinking?” (yes/no)
  ○ **Others concerned about drinking**: “Has a relative or friend, doctor or other health worker been concerned about your drinking or suggested that you cut down?” (yes/no)
- To assess proxies of alcohol dependence:
  ○ Using criteria from the International Classification of Diseases (WHO, 1993): “In the past year, when you drank alcohol, did you ever experience an unsteady gait? Fall over? Have difficulty speaking? Have shaking hands the next morning?” (yes/no for each item)
  ○ Using items similar to those from the AUDIT scale [4, 5]: “How often during the last year have you failed to do something that you wanted or needed to do because of your drinking?” (Never, occasionally, sometimes, often).

#### Analysis

A power calculation was not performed a priori for this study because it was secondary in nature and nested within the RCCS. The effective sample size was established by enrolment into the cohort. All men and women aged 15–49 years who consented to participate in RCCS survey activities and responded to the main alcohol question were retained in the analytic sample. This was a total of 18,700 participants out of the eligible population of 32,870. Thus, the participation rate in this study was 57%. Common reasons for non-participation were not different from what Grabowski et al., previously reported elsewhere, such as being away at the time of the survey, having out-migrated, being age ineligible, refusing to participate, etc. [18, 19]. All analyses were completed using STATA software package V15 [10]. We assessed overall prevalence and correlates of alcohol use in the Rakai region and also stratified all analyses by gender. The analysis was cross sectional and all independent variables were between subjects. First, we conducted chi-square analysis to assess differences in frequencies of sociodemographic characteristics, tobacco use, past year drug use and HIV status between men and women. All variables were statistically significantly different by gender, justifying the need for gender stratified analyses in our logistic regression models which we fit to calculate the unadjusted odds ratios (ORs) and 95% confidence intervals (CIs) to estimate associations between alcohol use and key socio-demographics, other drug use behaviors and HIV status. Next, we fit adjusted logistic regression models, controlling for covariates found to be significantly associated with alcohol use at the P<0.2 level in the bivariate analysis (i.e., unadjusted models). We used an alpha of 0.05 and two-tailed tests to determine statistical significance of observed associations reported in both bivariate and adjusted models. Six covariates were retained in the final models (age, religion, education, occupation, marital status, and household SES) due to their significance in bivariate analysis and existing literature suggesting they are known confounders. Occupation was excluded from the adjusted models assessing associations between community type and alcohol use since (as described above), a defining feature of community type was the primary occupation of its residents. Thus, we assumed occupation was in the causal pathway between community type and alcohol use.

Additional analysis of the sub-population of drinkers was conducted to describe the effects of drinking on alcohol users during the past year. Frequencies were assessed and differences were computed via chi-square analysis. Multicollinearity between the independent variables was checked with the Pearson’s correlation coefficient. A correlation coefficient close to +1 implied presence of multicollinearity between those two variables. We also ran the collinearity diagnostics after the adjusted logistic regression model. A Variance Inflation Factor (VIF) of 5 and above implied presence of multi-collinearity. In both gender stratified models, multicollinearity was ruled out because correlations between individual independent variables were weak and no VIFs exceeded two.

## Results

### Social and demographic characteristics of all participants

A total of 18,700 men and women were included in our analytic sample. Table 1 displays sociodemographic characteristics and alcohol and drug use behaviors for men and women, separately and overall. Just over half (54%) of the participants were women, 84% were Christian, 66% had a primary school education or less, and 57% were married at the time of interview. The largest proportion of participants (55%) were drawn from agrarian communities; 25% were from fishing villages and 20% from trading centers. The most commonly reported occupation among women was agriculture or housework (40%) followed by other or no work (29%) and 14% were shopkeepers or tradeswomen. Among men, the most common occupations were agricultural/domestic work (20%) and fishing (14%) with 19% of men identifying as students.

**Table 1:** Sociodemographic characteristics, past year drug use, HIV status, past year sexual activity and past year alcohol use and risk behaviors of men and women who participated in RCCS between March 2015 and September 2016 (N = 18,700), overall and by gender.

	Full sample n (%) N = 18700	Men n (%) N = 8690	Women n (%) N = 10010	Chi Square P-value for difference between men and women
**Age (years)**				
15–19	3230 (17.3)	1594 (18.3)	1636 (16.3)	<0.01
20–24	3219 (17.2)	1437 (16.5)	1782 (17.8)	
25–34	6226 (33.3)	2766 (31.8)	3460 (34.6)	
35+	6025 (32.2)	2893 (33.3)	3132 (31.3)	
**Religion**				
Christian	15657 (83.7)	7234 (83.2)	8423 (84.1)	<0.01
Muslim	2612 (14)	1214 (14)	1398 (14)	
Other/None	431 (2.3)	242 (2.8)	189 (1.9)	
**Education level**				
None/Primary	12376 (66.2)	6017 (69.2)	6359 (63.5)	<0.01
Secondary/higher	6324 (33.8)	2673 (30.8)	3651 (36.5)	
**Occupation**				
Agricultural/Housework	5698 (30.5)	1684 (19.4)	4014 (40.1)	<0.01
Bar/Restaurant	598 (3.2)	32 (0.4)	566 (5.7)	
Trucking	167 (0.9)	167 (1.9)	0 (0)	
Fishing	1194 (6.4)	1181 (13.6)	13 (0.1)	
Student	2822 (15.1)	1648 (19)	1174 (11.7)	
Trade/shop keeper	2378 (12.7)	998 (11.5)	1380 (13.8)	
Other/no occupation	5843 (31.2)	2980 (34.3)	2863 (28.6)	
**Marital status**				
Currently married	10684 (57.1)	4623 (53.2)	6061 (60.5)	<0.01
Previously married	2986 (16)	1056 (12.2)	1930 (19.3)	
Never married	5030 (26.9)	3011 (34.6)	2019 (20.2)	
**Household SES**				
High	11342 (60.7)	5018 (57.7)	6324 (63.2)	<0.01
Middle	3944 (21.1)	1873 (21.6)	2071 (20.7)	
Low	3414 (18.3)	1799 (20.7)	1615 (16.1)	
**Community type**				
Agrarian	10364 (55.4)	4772 (54.9)	5592 (55.9)	<0.01
Trading	4070 (21.8)	1698 (19.5)	2372 (23.7)	
Fishing	4266 (22.8)	2220 (25.5)	2046 (20.4)	
**HIV status**				
Negative	15208 (81.3)	7383 (85)	7825 (78.2)	<0.01
Positive	3492 (18.7)	1307 (15)	2185 (21.8)	
**Other drug use in past year**				
Marijuana	289 (1.5)	268 (3.1)	21 (0.2)	<0.01
Amphetamines	14 (0.1)	9 (0.1)	5 (0)	0.18
Aerofuels	11 (0.1)	4 (0)	7 (0.1)	0.5
Mayirungi	264 (1.4)	241 (2.8)	23 (0.2)	<0.01
Heroin	3 (0)	1 (0)	2 (0)	0.65
Other	38 (0.2)	36 (0.4)	2 (0)	<0.01
**Any drug use in past year**				
Yes	502 (2.7)	452 (5.2)	50 (0.5)	<0.01
No	18198 (97.3)	8238 (94.8)	9960 (99.5)	
**Tobacco smoker**				
Yes	1436 (7.7)	1167 (13.4)	269 (2.7)	<0.01
No	17264 (92.3)	7523 (86.6)	9741 (97.3)	
**Any past year alcohol use**				
Yes	10206 (54.6)	4138 (47.6)	6068 (60.6)	<0.01
No	8494 (45.4)	4552 (52.4)	3942 (39.4)	

HIV prevalence was 19% and was higher among women (22%) than men (15%). Past year smoking was reported by 8% of participants and prevalence of using any other illicit drugs (excluding alcohol) in the past year was low (3%), especially among women (0.5%) compared to 5% among men. Among the 502 participants who used (other) substances in the past year, the most commonly reported were marijuana (58%) and *mayirungi* (53%).

### Prevalence and correlates of alcohol use

#### Prevalence and correlates of alcohol use among all participants

Table 2 shows unadjusted and adjusted logistic regression output for all correlates of past year alcohol use among the total sample of RCCS participants. The population prevalence of past year alcohol use was 45% and women were significantly less likely to report its use, relative to men (aOR = 0.6, 96% CI 0.6, 0.6, p<0.01).

**Table 2:** Unadjusted and adjusted logistic regression models of correlates of alcohol use in the past 12 months in all men and women (N = 18,700) who participated in RCCS between March 2015 and September 2016.

	Unadjusted				Adjusted*			
	Odds Ratio	95% CI		P>z	Odds Ratio	95% CI		P>z
**Sex**				<0.01				<0.01
Males	Ref				Ref			
Females	0.6	(0.6,	0.6)		0.6	(0.5,	0.6)	
**Age group**				<0.01				<0.01
15–19	Ref				Ref			
20–24	2.8	(2.5,	3.2)		2.1	(1.8,	2.3)	
25–34	5.1	(4.6,	5.6)		3.0	(2.6,	3.4)	
≥ 35 years	6.1	(5.5,	6.8)		3.1	(2.7,	3.6)	
**Religion**				<0.01				<0.01
Christian	Ref				Ref			
Muslim	0.2	(0.2,	0.2)		0.1	(0.1,	0.1)	
Other or no religion	1.1	(0.9,	1.3)		0.7	(0.6,	0.9)	
**Education**				<0.01				0.07
Primary/None	Ref				Ref			
Secondary/Higher	0.7	(0.6,	0.7)		0.9	(0.9,	1.0)	
**Occupation**				<0.01				<0.01
Agricultural/Housework	Ref				Ref			
Bar/Restaurant	1.7	(1.4,	2.0)		1.6	(1.3,	2.0)	
Trucking	1.5	(1.1,	2.1)		1.0	(0.7,	1.5)	
Fishing	3.2	(2.8,	3.7)		1.4	(1.2,	1.7)	
Student	0.4	(0.3,	0.4)		0.7	(0.6,	0.8)	
Trade/Shop keeper	1.0	(0.9,	1.1)		0.9	(0.8,	1.0)	
Other/no occupation	1.0	(0.9,	1.1)		1.0	(0.9,	1.1)	
**Marital status**				<0.01				<0.01
Married	Ref				Ref			
Previously married	1.5	(1.4,	1.6)		1.3	(1.1,	1.4)	
Never	0.4	(0.3,	0.4)		0.7	(0.6,	1.7)	
**SES**				<0.01				<0.01
High	Ref				Ref			
Middle	1.1	(1.0,	1.1)		0.9	(0.9,	1.0)	
Low	1.5	(1.4,	1.6)		0.8	(0.7,	0.9)	
Residence**				<0.01				<0.01
Agrarian	Ref				Ref			
Trading	1.0	(0.9,	1.1)		1.1	(1.0,	1.1)	
Fishing	1.8	(1.7,	2.0)		1.6	(1.4,	1.7)	
**HIV status**				<0.01				0.09
Negative	Ref				Ref			
Positive	1.8	(1.7,	1.9)		1.1	(1.0,	1.2)	
**Drug use in past year**				<0.01				<0.01
Yes	Ref				Ref			
No	0.2	(0.2,	0.3)		0.4	(0.3,	0.5)	
**Tobacco smoker**				<0.01				<0.01
Yes	Ref				Ref			
No	0.2	(0.1,	0.2)		0.3	(0.3,	0.3)	

*Adjusted models controlled for age, religion, education, occupation, marital status, and household SES

**Adjusted model controlled for age, religion, education, marital status, and household SES

Looking at the total population by community type, alcohol use was reported by 57%, 42% and 41% of RCCS participants in fishing, agrarian and trade center areas, respectively. Compared to living in an agrarian area, living in a fishing community was significantly associated with increased odds of drinking (aOR = 1.6, 95% CI 1.4, 1.7, p<0.09) but some nuances emerged in these results when assessing influence of community type by gender.

In the unadjusted model, the odds of reporting past year alcohol use were higher among people living with HIV, compared to people not living with HIV (OR = 1.8, 95% CI 1.7, 1.9, p<0.01). In the adjusted model, however, this relationship was attenuated and no longer significant (aOR = 1.1, 95% CI 1.0, 1.2, p = 0.19).

Below we present findings on prevalence and correlates of past year alcohol use separately for men and women.

#### Prevalence and correlates of alcohol use among male participants

Past year alcohol use was reported by 52% of male participants, overall, and 64%, 49% and 46% of men in fishing, agrarian and trade center areas, respectively. Being a non-smoker, reporting no other drug use in the past year, being a student and having never been married were all independently associated with lower odds of past year alcohol use among men, even after controlling for potential confounders. Table 3 shows that other factors associated with increased odds of past year alcohol use among men included religion, marital status and older age.

**Table 3:** Unadjusted and adjusted logistic regression models of correlates of alcohol use in the past 12 months in all men (N = 8,690) who participated in RCCS between March 2015 and September 2016.

	Unadjusted				Adjusted*			
	Odds Ratio	95% CI		P>z	Odds Ratio	95% CI		P>z
**Age group**				<0.01				<0.01
15–19	Ref				Ref			
20–24	3.0	(2.6,	3.5)		2.3	(1.9,	2.7)	
25–34	6.1	(5.3,	7.0)		3.5	(2.8,	4.2)	
≥ 35 years	8.6	(7.5,	10.0)		4.2	(3.4,	5.2)	
**Religion**				<0.01				<0.01
Christian	Ref				Ref			
Muslim	0.1	(0.1,	0.2)		0.1	(0.1,	0.1)	
Other or no religion	1.1	(0.8,	1.4)		0.7	(0.5,	0.9)	
**Education**				<0.01				<0.20
Primary/None	Ref				Ref			
Secondary/Higher	0.7	(0.6,	0.7)		0.9	(0.8,	1.0)	
**Occupation**				<0.01				<0.01
Agricultural/Housework	Ref				Ref			
Bar/Restaurant	0.7	(0.4,	1.4)		0.9	(0.4,	1.9)	
Trucking	0.9	(0.7,	1.3)		1.1	(0.8,	1.5)	
Fishing	2.0	(1.7,	2.4)		1.9	(1.5,	2.4)	
Student	0.3	(0.2,	0.3)		0.7	(0.6,	0.8)	
Trade/Shop keeper	0.8	(0.7,	1.0)		0.9	(0.8,	1.1)	
Other/no occupation	0.8	(0.7,	0.9)		1.0	(0.9,	1.2)	
**Marital status**				<0.01				<0.01
Married	Ref				Ref			
Previously married	2.0	(1.7,	2.3)		1.7	(1.4,	2.0)	
Never	0.3	(0.3,	0.3)		0.7	(0.6,	0.9)	
**SES**				<0.01				0.36
High	Ref				Ref			
Middle	1.2	(1.1,	1.4)		1.0	(0.9,	1.2)	
Low	1.7	(1.5,	1.9)		0.9	(0.8,	1.1)	
Community type**				<0.01				<0.01
Agrarian	Ref				Ref			
Trading	0.9	(0.8,	1.0)		1.0	(0.9,	1.1)	
Fishing	1.8	(1.6,	2.0)		1.3	(1.2,	1.5)	
**Tobacco smoker**				<0.01				<0.01
Yes	Ref				Ref			
No	0.2	(0.2,	0.2)		0.3	(0.3,	0.4)	
**Drug use in past 12 months**				<0.01				<0.01
Yes	Ref				Ref			
No	0.3	(0.2,	0.3)		0.4	(0.3,	0.5)	
**HIV status**				<0.01				0.43
Negative	Ref				Ref			
Positive	2.5	(2.2,	2.8)		0.9	(0.8,	1.1)	

*Adjusted models controlled for age, religion, education, occupation, marital status, and household SES

**Adjusted model controlled for age, religion, education, marital status, and household SES

Living in a fishing community was strongly associated with men’s alcohol use, both in unadjusted models (OR = 1.8, 95% CI 1.6, 2.0, p<0.01) and after controlling for covariates (OR = 1.3, 95% CI 1.2, 1.5, p<0.01). Additionally, the adjusted odds of past year alcohol use were almost twice as high among men who worked in the fishing industry or as a fisherman, relative to agricultural workers (aOR = 1.9, 95% CI 1.5, 2.4, p<0.01).

Men’s HIV status was significantly associated with alcohol use in unadjusted models. Specifically, the odds of past year drinking were 2.5 times higher in men living with HIV, relative to HIV-negative men (OR = 2.5, 95% CI 2.2, 2.8, p<0.01). In multivariate adjusted models, however, this relationship was no longer significant (aOR = 0.9, 95% CI 0.8, 1.1, p = 0.43).

#### Prevalence and correlates of alcohol use among female participants

Past year alcohol use was reported by 39% of female participants, overall, and 50%, 36% and 38% of women in fishing, agrarian and trade center areas, respectively. Being a non-smoker, reporting no past year drug use, being a student, living in a low or middle SES household, and having never been married were all independently associated with lower odds of reporting past year alcohol use among women in unadjusted and adjusted models. Table 4 shows that other factors associated with increased odds of past year alcohol use among women included religion (i.e., being Christian vs. Muslim, another religion or no religion), increased age, marital status (i.e., being previously—as opposed to currently or never- married or in consensual union), working in a bar or restaurant and living in a fishing area.

**Table 4:** Unadjusted and adjusted logistic regression models of correlates of alcohol use in the past 12 months in all women (N = 10,010) who participated in RCCS between March 2015 and September 2016.

	Unadjusted				Adjusted*			
	Odds Ratio	95% CI		P>z	Odds Ratio	95% CI		P>z
**Age group**				<0.01				<0.01
15–19	Ref				Ref			
20–24	2.8	(2.4,	3.3)		1.8	(1.5,	2.2)	
25–34	4.6	(3.9,	5.3)		2.7	(2.2,	3.2)	
≥ 35 years	4.7	(4.0,	5.5)		2.6	(2.2,	3.2)	
**Religion**				<0.01				<0.01
Christian	Ref				Ref			
Muslim	0.2	(0.2,	0.3)		0.2	(0.2,	0.2)	
Other or no religion	0.9	(0.7,	1.2)		0.7	(0.56,	1.0)	
**Education**				<0.01				0.13
Primary/None	Ref				Ref			
Secondary/Higher	0.7	(0.7,	0.8)		0.9	(0.8,	1.0)	
**Occupation**				<0.01				<0.01
Agricultural/Housework	Ref				Ref			
Bar/Restaurant	2.1	(1.8,	2.5)		1.2	(1.3,	2.0)	
Fishing	1.2	(0.4,	3.6)		1.1	(0.3,	3.6)	
Student	0.3	(0.3,	0.3)		0.7	(0.6,	0.8)	
Trade/Shop keeper	1.1	(0.9,	1.2)		0.9	(0.8,	1.1)	
Other/no occupation	0.9	(0.9,	1.0)		1.0	(0.9,	1.2)	
**Marital status**				<0.01				<0.01
Married	Ref				Ref			
Previously married	1.4	(1.3,	1.5)		1.2	(1.0,	1.3)	
Never	0.3	(0.3,	0.4)		0.7	(0.6,	0.8)	
**SES**				<0.01				<0.01
High	Ref				Ref			
Middle	0.9	(0.8,	1.0)		0.9	(0.8,	1.0)	
Low	1.2	(1.1,	1.3)		0.7	(0.6,	0.8)	
Community type**				<0.01				<0.01
Agrarian	Ref				Ref			
Trading	1.1	(1.0,	1.2)		1.1	(1.0,	1.2)	
Fishing	1.8	(1.6,	2.0)		1.8	(1.6,	2.0)	
**Tobacco smoker**				<0.01				<0.01
Yes	Ref				Ref			
No	0.2	(0.2,	0.3)		0.3	(0.2,	0.4)	
**Drug use in past 12 months**				<0.01				<0.01
Yes	Ref				Ref			
No	0.3	(0.1,	0.5)		0.3	(0.2,	0.7)	
**HIV status**				<0.01				0.11
Negative	Ref				Ref			
Positive	1.7	(1.5,	1.8)		1.1	(1.0,	1.2)	

*Adjusted models controlled for age, religion, education, occupation, marital status, and household SES

**Adjusted model controlled for age, religion, education, marital status, and household SES

Similar to our findings from male participants, women’s HIV status was strongly linked with past year alcohol use in unadjusted models (OR = 1.7, 95% CI 1.5, 1.8, p<0.01) but after controlling for confounders this relationship was no longer significant (aOR = 1.1, 95% CI 1.0, 1.2, p = 0.11).

#### Consequences associated with alcohol consumption among past year drinkers

Table 5 shows that overall, a greater proportion of men reported affirmatively to experiencing every factor related to alcohol use. Alcohol-related aggression and violence were reported by 16% and 11% of participants who indicated they became angry and/or violent (respectively) while drinking in the past year. Slightly more than half of all participants who reported past year alcohol use (54.8%) said they never felt a need to cut down on their drinking, while one-fifth (19.5%) reported they “often” felt a need to reduce their drinking. More men than women reported drinking on the job (28.5% versus 15.9% of women, p<0.01).

**Table 5:** Consequences of alcohol consumption among men and women who participated in RCCS between March 2015 and September 2016 and reported past year drinking.

	Total (N = 8,494)	Men (n = 4,552)	Women (n = 3,942)	p-value
	n (%)	n (%)	n (%)	
**ALCOHOL-RELATED AGGRESSION AND VIOLENCE**				
*In the past year when you drank alcohol*, *did you ever*:				
Get angry				<0.01
Yes	1394 (16.4)	964 (21.2)	430 (10.9)	
No	7100 (83.6)	3588 (78.8)	3512 (89.1)	
Get violent / in a fight				<0.01
Yes	909 (10.7)	602 (13.2)	307 (7.8)	
No	7585 (89.3)	3950 (86.8)	3635 (92.2)	
**HARMFUL ALCOHOL USE**				
*In the past year when you drank alcohol*, *did you ever*:				
Feel ashamed of something done while drinking?				<0.01
Yes	1016 (12.0)	729 (16.0)	287 (7.3)	
No	7478 (88.0)	3823 (84.0)	3655 (92.7)	
Forget things you did or that happened while drinking?				<0.01
Yes	984 (11.6)	743 (16.3)	241 (6.1)	
No	7510 (88.4)	3809 (83.7)	3701 (93.9)	
Has a relative, friend, doctor, or other health worker been concerned about your drinking or suggested you cut down?				<0.01
Yes	2047 (24.1)	1454 (31.9)	593 (15.0)	
No	6446 (75.9)	3097 (68.1)	3350 (85.0)	
**ALCOHOL DEPENDENCE**				
*In the past year when you drank alcohol*, *did you ever*:				
Have an unsteady gait?				<0.01
Yes	2180 (25.7)	1608 (35.3)	572 (14.5)	
No	6314 (74.3)	2944 (64.7)	3370 (85.5)	
Fall over?				<0.01
Yes	262 (3.1)	192 (4.2)	70 (1.8)	
No	8232 (96.9)	4360 (95.8)	3872 (98.2)	
Have difficulty speaking?				<0.01
Yes	664 (7.8)	489 (10.7)	175 (4.4)	
No	7830 (92.2)	4063 (89.3)	3767 (95.6)	
Have shaking hands the next morning?				<0.01
Yes	640 (7.5)	488 (10.7)	152 (3.9)	
No	7854 (92.5)	4064 (89.3)	3790 (96.1)	
Fail to do something you wanted/needed to do?				<0.01
Never	6703 (78.9)	3189 (70.1)	3514 (89.1)	
Occasionally	1242 (14.6)	981 (21.6)	262 (6.6)	
Sometimes	373 (4.4)	271 (6.0)	102 (2.6)	
Often	176 (2.1)	111 (2.4)	65 (1.6)	
**LOSS OF CONTROL RELATED TO DRINKING**				
*How often during the last year have you felt you should* *cut down on your drinking or stop altogether*?				<0.01
Never	4654 (54.8)	2278 (50)	2376 (60.3)	
Occasionally	1360 (16.0)	895 (19.7)	466 (11.8)	
Sometimes	827 (9.7)	522 (11.5)	305 (7.7)	
Often	1653 (19.5)	857 (18.8)	796 (20.2)	
**ALCOHOL’S IMPACT ON DAILY RESPONSIBILITIES AND ACTIVITIES**				
*In the past year have you taken alcohol while at work*?				<0.01
Yes	1924 (22.7)	1296 (28.5)	628 (15.9)	
No	6570 (77.3)	3256 (71.5)	3315 (84.1)	

Suggestive of harmful alcohol use, more men than women who reported past year alcohol use also reported feeling ashamed of something done while drinking (16% vs. 7%, p<0.01) and forgetting things they did or that happened while drinking (16% vs. 7%, p<0.01). Roughly one-fourth of past year alcohol users (24.1%) said a friend, doctor or other healthcare provider had suggested they cut down on their drinking, with more men than women responding “yes” to that question (31.9% versus 15%, p<0.01). In response to alcohol dependence-related questions, the most commonly reported sign of dependence was “unsteady gait” which was indicated by one-fourth of participants (35.3% of men and 14.5% of women). One-fifth of past year alcohol users reported that in the past year when they drank alcohol, they failed to do something they wanted/needed to do, occasionally (15%), sometimes (4%), often (2%).

## Discussion

Slightly less than half (45%) of all participants in Rakai said they drank alcohol in the past year, with significantly more men than women reporting its consumption. The past year prevalence of alcohol use in Rakai was significantly higher in fishing communities, compared to agrarian areas. These results are consistent with findings from other regions of Uganda where alcohol use was recorded to be higher in men than women [3, 20, 21] and in fishing areas, relative to other community types [21].

Irrespective of gender, older age was correlated with higher levels of past year alcohol use, with the odds of past year alcohol use trending upwards from the youngest to oldest age brackets. Being married (relative to divorced, separated or widowed), identifying as Muslim, and reporting no cigarette smoking or past year use of other drugs were all significantly associated with lower odds of past year alcohol use among both men and women in Rakai.

Among men in our sample, those who earned their primary income through fishing had the highest odds of past year alcohol use compared to men in the reference occupation of agriculture (aOR = 1.9, 95% CI 1.5, 2.4, p < 0.01). This builds on our previous qualitative research with residents from Rakai fishing communities who explained that fishing is the most lucrative local industry, providing ample disposable income that can be (and is) used to purchase alcohol [22] in any location. Similarly, men in fishing areas were significantly more likely to report past year alcohol use, relative to men in agrarian communities. These findings add to a growing body of literature suggesting rates of both alcohol consumption and AUDs are high in Uganda’s fishing communities along Lake Victoria [9, 21, 23].

The adjusted odds of women’s past year alcohol use was also statistically significantly different by community type. Women in fishing areas were 1.7 times more likely to report past year alcohol use than women in agrarian communities, even after controlling for covariates (aOR = 1.8 95% CI 1.6, 12.0, p < 0.01). These differentials in trends of women’s alcohol use by location type are important, even in light of the finding that women were significantly less likely to drink overall, compared to men. It is widely agreed that most gender differences in drinking patterns in Uganda are rooted in social and cultural norms and beliefs. While acceptable for Ugandan men to drink in bars and other public places, with friends and workmates, both in and outside their own community, social norms dictate women should typically only drink at home/in their own community, and with friends or family members, including their husband or sexual partner [24]. In our own qualitative research, participants confirmed it was less acceptable for women to drink (compared to men) in the Rakai region, but these norms were different when it came to life in or near fishing communities. It was commonly perceived that fishing areas were unique due to the high density of bars/drinking establishments in these areas, and a correlated high likelihood of women working in or around these establishments [22]. The current study found women’s occupation to be statistically significantly associated with past alcohol use, with higher odds of drinking reported among women who worked at a bar/restaurant. This finding supports our previous qualitative research in which community members narrated how women working in/around bars were more likely to use alcohol, relative to women in other professions and settings (e.g., agrarian communities) [22]. Thus, it makes sense that women in fishing areas had heightened likelihood of drinking given their tendency to do bar work and because so many men in fishing areas, many of whom were presumably the partners of women interviewed, were also drinking.

Our findings on consequences of alcohol use among current drinkers align with global evidence suggesting men are not only more likely to drink but they are more likely (than women) to cause and experience adverse alcohol-related outcomes [25]. In the current study, all negative alcohol-related consequences were reported by a greater proportion of men than women. For instance, 35% of men reported an unsteady gait, 11% reported difficulty speaking and shaking hands the next morning, both of which suggest heavy drinking and/or alcohol dependence. Similarly, a higher proportion of men (29%) than women (16%) reported using alcohol on the job in the past year, suggesting alcohol’s’ impact on daily responsibilities and activities. Overall, these findings highlight men as a priority population for interventions aimed at reducing hazardous or harmful alcohol use in this context.

Although HIV status was not associated with past year alcohol use, after controlling for potential confounders, among RCCS participants, a strong relationship emerged between drinking and living with HIV infection in our unadjusted models. These differences imply that one or more covariates were associated with both HIV status and alcohol use in our sample and do *not* minimize the importance of addressing this relationship. In adjusted models for both men and women, age group and marital status were associated with the highest levels of confounding. We previously found alcohol use before sex increased risk for HIV incidence in men and women [10, 11] and physical and sexual violence among young women in Rakai [10, 11]. Further research is warranted to better understand the determinants of alcohol use in sexual situations, how drinking in this context increases risk for HIV (and other sexually transmitted) infection and if modifiable factors exist for potential harm reduction interventions.

This study had limitations to note. First, our data were cross-sectional, precluding our ability to establish temporality or causality in the relationships between alcohol use and the variables assessed. Second, alcohol and drug use behaviors were self-reported and participants might have underestimated or forgotten their true behaviors, potentially leading to reduced accuracy of findings. Additionally, social desirability bias could have influenced participants to intentionally under-report drinking, drug use and other stigmatized behaviors. Third, our study had a very large sample size which increased our ability to find statistically significant associations where they existed even when the magnitude of the association was relatively small. For this reason, significant results should be interpreted with caution and the magnitude of the association should be considered when interpreting the public health implications of our findings. At the same time, however, the large sample size enabled us to capture a diverse cross-section of the Rakai community, increasing the generalizability of our findings. Finally, and perhaps most substantial is that the alcohol use measures in RCCS are limited. They do not assess frequency of drinking or volume of alcohol consumed, limiting our understanding of participants’ full range of drinking patterns and potentially impacting the way in which we were able to assess relationships between alcohol use and other key outcomes, such as HIV status. Further, although some measures included in our analysis were based on AUDIT items, the questions were not taken word for word (from AUDIT) and the full tool for identifying AUDs was not included in RCCS. Thus, we might not have fully captured the different aspects of severity or intensity of alcohol use, nor could we accurately assess relationships between key sociodemographic variables and levels of drinking risk. We recommend future research in this population, using AUDIT or another standardized validated psychometric tool for alcohol use, as well as an alcohol biomarker to increase the validity of the measurement. Our study also had strengths. The data used derived from a population-based cohort in a mature, generalized HIV epidemic and included a large and representative sample of Rakai residents from multiple community types, lending generalizability to our findings, conclusions and suggestions. The research presented in this paper fills an important gap in the literature by establishing prevalence and correlates of alcohol use in Rakai, Uganda, a region where previous work has identified alcohol use and HIV as interrelated and synergistic public health issues. Exploring associations between these two factors is important for public health planning. Alcohol use is also associated with a myriad of other poor health outcomes and it is critical to identify which populations should be targeted for alcohol use intervention programming.

## Conclusions

Alcohol use differed by gender, community type and occupation. Being male, living in a fishing community and working in the fishing or restaurant/bar industry were associated with higher odds of drinking and men reported more negative alcohol-related consequences than women. Cultural norms and high cash incomes of fishermen may partially explain these findings which suggest alcohol reduction programs should be implemented in Uganda’s fishing communities and people working in high risk occupations, particularly men, should be targeted for participation and retention in alcohol reduction interventions.
